# Chewing areca nut increases the risk of coronary artery disease in taiwanese men: a case-control study

**DOI:** 10.1186/1471-2458-12-162

**Published:** 2012-03-07

**Authors:** Wei-Chung Tsai, Ming-Tsang Wu, Guei-Jane Wang, Kun-Tai Lee, Chien-Hung Lee, Ye-Hsu Lu, Hsueh-Wei Yen, Chih-Sheng Chu, Yi-Ting Chen, Tsung-Hsien Lin, Ho-Ming Su, Po-Chao Hsu, Kai-Hung Cheng, Tsai-Hui Duh, Ying-Chin Ko, Sheng-Hsiung Sheu, Wen-Ter Lai

**Affiliations:** 1 Division of Cardiology, Department of Internal Medicine, Kaohsiung Medical University Hospital, Kaohsiung Medical University, Kaohsiung, Taiwan; 2 Department of Internal Medicine, Kaohsiung Municipal Hsiao-Kang Hospital, Kaohsiung Medical University, Kaohsiung, Taiwan; 3 Department of Family Medicine, Kaohsiung Medical University Hospital, Kaohsiung, Taiwan; 4 Department of Public Health, Kaohsiung Medical University, Kaohsiung, Taiwan; 5 Center of Environmental and Occupational Medicine, Kaohsiung Municipal Hisao-Kang Hospital, Kaohsiung, Taiwan; 6 National Research Institute of Chinese, Taipei, Taiwan; 7 Department of Public Health, Kaohsiung Medical University, Kaohsiung, Taiwan; 8 Department of Pathology, Kaohsiung Medical University Hospital, Kaohsiung Medical University, Kaohsiung, Taiwan; 9 Department of Medicinal and Applied Chemistry, Kaohsiung Medical University, Kaohsiung, Taiwan; 10 Center of Excellence for Environmental Medicine, Kaohsiung Medical University, Kaohsiung, Taiwan

**Keywords:** Areca nut, Coronary artery disease, Atherosclerosis

## Abstract

**Background:**

Areca nut chewing has been reported to be associated with obesity, metabolic syndrome, hypertension, and cardiovascular mortality in previous studies. The aim of this study was to examine whether chewing areca nut increases the risk of coronary artery disease (CAD) in Taiwanese men.

**Methods:**

This study is a hospital-based case-control study. The case patients were male patients diagnosed in Taiwan between 1996 and 2009 as having a positive Treadmill exercise test or a positive finding on the Thallium-201 single-photon emission computed tomography myocardial perfusion imaging. The case patients were further evaluated by coronary angiography to confirm their CAD. Obstructive CAD was defined as a ≥ 50% decrease in the luminal diameter of one major coronary artery. The patients who did not fulfill the above criteria of obstructive CAD were excluded.

The potential controls were males who visited the same hospital for health check-ups and had a normal electrocardiogram but no history of ischemic heart disease or CAD during the time period that the case patients were diagnosed. The eligible controls were randomly selected and frequency-matched with the case patients based on age. Multiple logistic regression analyses were used to estimate the odds ratio of areca nut chewing and the risk of obstructive CAD.

**Results:**

A total of 293 obstructive CAD patients and 720 healthy controls, all men, were analyzed. Subjects who chewed areca nut had a 3.5-fold increased risk (95% CI = 2.0-6.2) of having obstructive CAD than those without, after adjusting for other significant covariates. The dose-response relationship of chewing areca nut and the risk of obstructive CAD was also noted. After adjusting for other covariates, the 2-way additive interactions for obstructive CAD risk were also significant between areca nut use and cigarette smoking, hypertension and dyslipidemia.

**Conclusions:**

Long-term areca nut chewing was an independent risk factor of obstructive CAD in Taiwanese men. Interactive effects between chewing areca nut and cigarette smoking, hypertension, and dyslipidemia were also observed for CAD risk. Further exploration of their underlying mechanisms is necessary.

## Background

Ischemic heart disease, including coronary artery disease (CAD), is the leading cause of death worldwide [1]. CAD patients have a high risk of myocardial infarction, heart failure, angina pectoris, stroke, and even sudden cardiac death [2]. In Taiwan, cardiovascular disease was the second leading cause of death after cancer in 2010. Cardiovascular disease accounted for 10.8% of the total deaths in the Taiwanese population [3].

Areca nut chewing is the fourth most popular substance abuse habit in the world [4]. Previous studies have found that chewing areca nut is associated with obesity, metabolic syndrome, hypertension (HTN), and all-cause mortality [5-9]. A few epidemiological studies also linked areca nut chewing with the risk of cardiovascular disease [9-11]. Although those studies were conducted in the community, their diagnosis of cardiovascular disease was based on a questionnaire or the report of the International Classification of Disease, Ninth Revision (ICD-9) codes 390-459. These codes were not reconfirmed by cardiologists and likely introduced random misclassification of the outcome of interest. In addition, the exposure of interest (areca nut chewing) was also collected from a non-validated questionnaire. Since the habit of chewing areca nut is prevalent in Taiwanese men, we used the accurate method of diagnosed obstructive CAD to further examine the relationships between areca nut chewing and the risk of CAD in a Taiwanese male population.

## Methods

### Study population

This study is a hospital-based case-control study. The case patients were male patients diagnosed as having a positive treadmill exercise test or a positive finding on the Thallium-201 (Tl-201) single-photon emission computed tomography (SPECT) myocardial perfusion imaging (MPI) at the Kaohsiung Medical University Hospital (KMUH), a medical center in the southern Taiwan, between 1996 and 2009. The positive Treadmill exercise test was defined as an ST segment elevation or depression ≥ 1 mm in three consecutive beats in ≥ 2 consecutive leads. A positive finding on the Tl-201 SPECT MPI was defined as reversible defects in at least one vascular territory. Case patients were also performed a coronary angiography (CAG) using the Judkins technique. Then, the angiograms were analyzed by two experienced cardiologists who were blinded to the results of the questionnaire. The severity of stenosis was assessed by the quantitative coronary angiography (Infinix, Celeve CBi-BP, XTP-8100 G/DFP-8000D, Toshiba Medical Systems Corp) of the major coronary arteries and their major branches. Obstructive CAD was defined as a ≥ 50% decrease in the luminal diameter of one major coronary artery [12,13]. If an artery had more than one significant stenosis, we analyzed only the most severe lesion. In the cases of discrepancies between the two cardiologists, a third cardiologist was consulted to determine the final diagnosis. Case patients who did not fulfill the above criteria were diagnosed as having non-obstructive CAD (n = 30) and were excluded from the study.

The potential controls were males who visited the same hospital for health check-ups during the same time period as the case patients. The potential controls had a normal electrocardiogram record during their examination and did not have any history of ischemic heart disease or CAD. The eligible controls were randomly selected and frequency-matched with the case patients based on age (≤ 40, 41-60, and > 60 years old). In total, 293 case patients and 720 healthy controls were included in this study. This study was approved by the ethics committee of KMUH, and all study subjects provided written informed consent.

### Questionnaire

A standardized questionnaire was used by a well trained research assistant to interview study subjects. The questionnaire collected detailed information about the subjects' demographic characteristics and history of using three major substances (cigarette smoking, alcohol consumption, and areca nut chewing). The detailed information has been described elsewhere [14,15]. In brief, cigarette smokers were defined as regular consumers of 10 cigarettes or more per day for > 6 months. Alcohol drinkers were defined as regular consumers of alcoholic drinks more than once a week for > 6 months. Areca nut chewers were defined as regular consumers of one or more pieces of areca nut per day for > 6 months. If the study subject was a regular substance user, then detailed questions about the time of start and quit, daily amount used, and type of consumption were also collected. Former users were those who had stopped using the substance for ≥ 1 year.

Two kinds of chewing areca nut commonly used in Taiwan were also included on the questionnaire: Lao-hwa and betel leaf. The composite of the Lao-hwa regimen includes areca nut, betel palm inflorescence, and slaked lime, whereas the composite of betel leaf regimen includes areca nut, betel leaf, and slaked lime [16].

The items on the questionnaire regarding the use of these three major substances were validated in our previous study [14,15]. In brief, we used different biomarkers in different specimens to verify information about cigarette smoking, alcohol, and areca nut chewing from questionnaire [14]. For verifying areca nut chewing, since areca nuts in Taiwan contain a high concentration of safrole, a carcinogen, our research collaborators analyzed safrole-DNA adducts using the P-postlabelling method in 47 tissue specimens of esophageal cancer (16 areca chewers and 31 non-areca chewers by questionnaire). Safrole-DNA adducts were detected in 5 (31.3%) out of 16 areca chewers and, in contrast, detected in none of the 31 non-areca chewers (Fischer's exact test, *p *= 0.0028) [14].

We quantified the cumulative amount of substance use by "drink-years" for alcohol consumption and "pack-years" for cigarette smoking and areca nut chewing. The number of "drink-years" and "pack-years" were calculated by multiplying the amount of the substances consumed per day, such as 15.75 g-alcohol drinks (one ~350 cc bottle of beer with 4.5% alcohol concentration), 20-cigarette packs or 10-areca nut packs, by the years of substance use.

Personal disease histories including hypertension (HTN), diabetes mellitus (DM), dyslipidemia, stroke, CAD, and arrhythmia were also recorded. HTN was defined as having a history of HTN or anti-HTN drug use. DM was defined as having a history of DM, using an oral glucose-lowering agent or insulin, or having a fasting blood sugar greater than 126 mg/dL. Dyslipidemia was defined as having a history of dyslipidemia, using a lipid-lowering agent or having an abnormal high level of plasma cholesterol (>200 mg/dL) during health exams.

### Statistical analysis

Demographic characteristics, histories of substance use and prevalence of disease were compared between healthy controls and case patients (obstructive CAD patients) by Chi-Square test or Fisher's exact test when appropriate. Because our main study aim was to investigate whether chewing areca nut is a risk factor for CAD, we used unconditional logistic regression to analyze the effect of different levels of areca nut use on CAD risk, after adjusting for other covariates that were statistically significant in the crude analyses. These covariates included body mass index (BMI), educational level, age, cigarette smoking, alcohol consumption, DM, HTN, and dyslipidemia.

To assess the interactive effect of areca nut use and other main risk factors (cigarette smoking, DM, HTN, or dyslipidemia) for CAD among subjects, Rothman's synergism index (SI) was used to assess the empirical deviation from the additive interaction relation [17]. A computed SI value that departed from the expected additive null was considered to be an additive interaction effect. The ORs and their variance-covariance matrix were used to calculate values of SI and their 95% CIs [17]. Statistical significance was defined by a *p *value less than 0.05. All statistical analyses were carried out by using SAS software (version 8.0; SAS Institute Inc, Cary, NC).

## Results

The percentages of CAD patients in the ≤ 40, 41-60, and > 60 year old age groups were 3.4% (10/293), 37.2% (109/293) and 59.4% (174/293), respectively. These percentages were similar to those of the healthy controls. Compared to healthy controls, the obstructive CAD patient groups had a significantly higher percentage of DM, HTN, dyslipidemia, cigarette smoking, and areca nut chewing. In addition, obstructive CAD patients were less educated, and more likely to be obese than the healthy controls (Table 1).

**Table 1:** Comparison of demographic and clinical characteristics in study subjects

Parameter	Healthy control		Obstructive CAD		*p *value^*a*^
Total	720		293		
Age (yrs)					
≤ 40	30	(4.2)	10	(3.4)	0.063
41-60	321	(44.6)	109	(37.2)	
> 60	369	(51.2)	174	(59.4)	
Educational levels					< 0.001
< High school	131	(18.2)	89	(30.4)	
High school	312	(43.3)	123	(42.0)	
> High school	277	(38.5)	81	(27.6)	
BMI					< 0.001
≤ 18.5	19	(2.6)	5	(1.7)	
18.5-27	591	(82.1)	197	(67.2)	
> 27	110	(15.3)	91	(31.1)	
DM					< 0.001
No	638	(88.6)	178	(60.8)	
Yes	82	(11.4)	115	(39.2)	
HTN					< 0.001
No	576	(80.0)	74	(25.3)	
Yes	144	(20.0)	219	(74.7)	
Dyslipidemia					< 0.001
No	610	(84.7)	89	(30.4)	
Yes	110	(15.3)	204	(69.6)	
Cigarette smoking (pack-years)					< 0.001
Never user	414	(57.5)	76	(25.9)	
User	306	(42.5)	217	(74.1)	
1-20	133	(18.5)	63	(21.5)	
> 20	173	(24.0)	154	(52.6)	
Alcohol consumption (drink-years)					0.078
Never user	518	(71.9)	191	(65.2)	
User	202	(28.1)	102	(34.8)	
1-20	100	(13.9)	46	(15.7)	
> 20	102	(14.2)	56	(19.1)	
Areca nut chewing (pack-years)					< 0.001
Never user	663	(92.1)	205	(70.0)	
User	57	(7.9)	88	(30.0)	
1-20	34	(4.7)	30	(10.2)	
> 20	23	(3.2)	58	(19.8)	

BMI, body mass index; CAD, coronary artery disease; CI, confidence interval; DM, diabetes mellitus; HTN, hypertension;

^***a***^Chi-Square test or Fisher's exact test when appropriate

After adjusting for age, educational levels, BMI, DM, HTN, dyslipidemia, cigarette smoking, and alcohol consumption, subjects who had ever chewed areca nut had an increasing risk (adjusted OR = 3.5, 95% CI = 2.0-6.2) of having obstructive CAD (Table 2). The significant results were similar in both former and current areca nut users. A dose-response relationship between cumulative areca nut exposure and obstructive CAD risk was also noted. Subjects who chewed Lao-hwa had a relatively higher risk of obstructive CAD (adjusted OR = 5.1; 95% CI = 1.9-13.6), compared to those chewing betel leaf (adjusted OR = 2.7; 95% CI = 1.3-5.7) (Table 2).

**Table 2:** Odds ratio for obstructive coronary artery disease associated with areca nut use

Total	Healthy controls (n = 720)	Obstructive CAD (n = 293)	Crude OR (95% CI)		**Adjusted OR (95% CI)**^***a***^	
	**N (%)**	**N (%)**				

Areca nuts						

Never-user	663 (92.1)	205 (70.0)	1.0		1.0	

User	57 (7.9)	88 (30.0)	5.9	(3.5-7.2)	3.5	(2.0-6.2)

Former user	34 (4.7)	69 (23.5)	2.6	(1.4-5.0)	3.0	(1.3-7.6)

Current user	23 (3.2)	19 (6.5)	6.6	(4.2-10.2)	3.8	(2.0-7.2)

Daily uses (pieces/day)						

1-20	37 (5.1)	36 (12.3)	3.1	(1.9-5.1)	2.0	(1.0-3.9)

> 20	20 (2.8)	52 (17.7)	8.4	(4.9-14.4)	7.5	(3.4-16.5)

Cumulative uses (pack-years)						

1-20	34 (4.7)	30 (10.2)	2.9	(1.7-4.8)	2.0	(1.0-4.2)

> 20	23 (3.2)	58 (19.8)	8.2	(4.9-13.6)	6.1	(2.9-12.7)

Type of uses ^*b,*^						

Betel leaf	36 (5.0)	36 (12.3)	3.2	(2.0-5.3)	2.7	(1.3-5.7)

Lao-hwa	11 (1.5)	26 (8.9)	7.6	(3.7-15.7)	5.1	(1.9-13.6)

Both^*c*^	7 (1.0)	23 (7.8)	10.6	(4.5-25.1)	3.7	(1.3-10.8)

BMI, body mass index; CAD, coronary artery disease; CI, confidence interval; OR, odds ratio;

^*a*^Adjusting for diabetes, hypertension, dyslipidemia, alcohol drink, cigarette smoking, age, educational levels, and BMI

^*b*^Six missing data

^*c *^Lao-hwa and Betel leaf

Because most of the male subjects who chewed areca nut also smoked cigarettes in Taiwan, we further restricted the analyses to the group of male subjects who smoked cigarettes. Compared to male subjects who only smoked cigarettes, those who both regularly smoked and chewed areca nuts had 2.2- (95% CI = 1.1-5.0) and 8.0-fold higher risks (95% CI = 3.6-17.9) of CAD for 1-20 pack-years and > 20 pack-years of areca nut chewing, respectively (Additional file 1: Table S1).

The 2-way synergistic effects between areca nut chewing and cigarette smoking, DM, HTN, and dyslipidemia on CAD risk among the subjects were evaluated in additive interaction models (Table 3). The expected OR were 4.3, 5.2, 11.9, and 15.9 when regarding the joint effects for combining areca nut using with smoking, DM, HTN and dyslipidemia for CAD, respectively. Based on the model of addictive scale, significant additive effects of areca nut chewing on CAD risk were found with cigarette smoking, HTN, and dyslipidemia (SI = 4.3 (95% CI = 1.3-13.7), 4.1 (95% CI = 1.7-9.7) and 2.9 (95% CI = 1.1-7.7) respectively). Besides uses of areca nut, the variables of DM, HTN, dyslipidemia and smoking were also the important risk factors of CAD in this study (Additional file 2: Table S2 and Additional file 3: Table S3).

**Table 3:** The additive interaction between conventional risk factors and areca nuts use for coronary artery disease

Factors/Category	Healthy controls (n = 720) N (%)	Obstructive CAD (n = 293) N (%)	**Adjusted OR (95% CI)**^*c*^		**Additive interaction**^***a,b***^	
					**EOR**^***a***^	SI^*b *^(95% CI)
Smoke/Areca nuts use						
Never/Never	410 (56.9)	73 (24.9)	1.0			
Ever/Never	253 (35.1)	132 (45.1)	3.8	(2.4-6.1)		
Never/Ever	4 (0.6)	3 (1.0)	1.5	(0.2-13.5)		
Ever/Ever	53 (7.4)	85 (29.0)	15.0	(7.8-28.9)	4.3	4.3 (1.3-13.7)
Diabetes/Areca nuts use						
Never/Never	591 (82.1)	128(43.7)	1.0			
Ever/Never	72 (10.0)	77 (26.3)	2.6	(1.5-4.3)		
Never/Ever	47 (6.5)	50 (17.1)	3.6	(1.9-6.9)		
Ever/Ever	10 (1.4)	38 (12.9)	10.4	(4.1-26.3)	5.2	2.2 (0.7-6.8)
Hypertension/Areca nuts use						
Never/Never	528(73.3)	51(17.4)	1.0			
Ever/Never	135(18.8)	154(52.6)	9.7	(6.0-15.6)		
Never/Ever	48 (6.7)	23 (7.8)	3.2	(1.5-6.5)		
Ever/Ever	9 (1.3)	65 (22.2)	45.5	(19.0-109.2)	11.9	4.1 (1.7-9.7)
Dyslipidemia/Areca nuts use						
Never/Never	560(77.8)	53(18.1)	1.0			
Ever/Never	103(14.3)	152(51.9)	13.0	(8.2-20.6)		
Never/Ever	50 (6.9)	36 (12.3)	3.9	(2.0-7.5)		
Ever/Ever	7 (1.0)	52 (17.7)	44.4	(17.1-115.2)	15.9	2.9 (1.1-7.7)

BMI, body mass index; CAD, coronary artery disease; CI, confidence interval; OR, odds ratio;

^*a*^Expected odds ratios estimated based on additive interaction models. ^*b*^Synergism index estimated by an additive interaction model. ^*c*^Odds ratios were adjusted for age, educational levels, BMI, alcohol drinking, and diabetes, hypertension, dyslipidemia, or cigarette smoking

## Discussion

The present study mainly found that chewing areca nut increased the risk of obstructive CAD in Taiwanese men, particularly among subjects who smoked cigarettes. Several previous studies have reported the association between areca nut chewing and the risk of cardiovascular diseases in Taiwan [8-10,18]. Although two of the four epidemiological studies were prospective cohort designs [8,10], their exposure and outcome of interest were from a questionnaire and ICD-9 codes, respectively, neither of which were validated by the authors. In addition, a variety of cardiovascular diseases, such as hypertensive heart disease, cardiomyopathy, ischemic heart disease and arrhythmia, were included and analyzed as the same disease in these studies.

Lan et al. first reported that in an elderly population, people who ever chewed areca nut were at a higher risk of all-cause (hazard ratio (HR) = 1.19, 95% CI: 1.05, 1.35) and cerebrovascular disease mortality (HR = 1.66, 95% CI: 1.19, 2.30), compared with those who never chewed areca nut [8]. In the same year, Guh et al. found that the OR for prevalent heart disease and a betel-quid consumption rate of 10 times/day was 1.37 (95% CI = 1.1-1.6) among women [18]. Subsequently, a study conducted by Lin et al. also found that former and current betel nut chewers had an increased risk for cardiovascular and all-cause mortality [9]. The former betel nut chewers had adjusted relative risk (RR) 1.56 (95% CI = 1.02-2.38) and 1.40 (95% CI = 1.17-1.68) for CVD and all-cause mortality respectively, when compared with never chewer. The current chewers had RR 2.02 (95% CI = 1.31-3.13) and 1.40 (95% CI = 1.16-1.70) for CVD and all-cause mortality respectively, when compared with never chewer. Yen et al. also found an independent dose-response effect between chewing betel nut and an increasing risk of incident CVD among men [10]. The betel-quid ever chewers were at higher risk of CVD (HR = 1.24, 95% CI = 1.11-1.39) when compared with never chewer.

To minimize outcome misclassification, the present study used CAG to confirm the diagnosis of obstructive CAD. In addition, the exposure of interest (areca nut chewing) in this study was validated in our previous research [14]. The prevalence of areca nut chewing was 7.9% in the control group in our study, which was compatible with the corresponding figures (~10%) in the previous survey [19].

We also found a dose-dependent relationship between areca nut chewing and CAD risk, as a higher amount of areca nut chewing was associated with a higher risk of obstructive CAD. The additive interactions in the risk of obstructive CAD between "areca nut chewing and cigarette smoking," "areca nut chewing and HTN" and "areca nut chewing and dyslipidemia" were also observed. CAD, which is similar to other atherosclerotic diseases, has been previously associated with risk factors such as DM, HTN, dyslipidemia, age and obesity [20]. Areca nut chewing has been previously reported to be associated with many risk factors of CAD. In one study, general and central obesity were related to areca nut chewing in Chinese males [5]. Areca nut chewing has also been associated with HTN in Taiwanese patients with type-2 DM [7]. The association between metabolic syndrome and areca nut chewing has also been reported [6,21]. Indians living in the United Kingdom were found to have high incidences of cardiovascular disease, HTN and late onset diabetes [22]. Those findings, along with ours, suggest that areca nut might act as an independent risk factor for CAD or as a mediator that modifies the risk of HTN or dyslipidemia.

The present study found that the Lao-hwa regimen was more potent than the betel leaf regimen by comparing the ORs for their associations with obstructive CAD. Betel leaf may have some beneficial effects for cardiovascular disease through its vasorelaxation, antioxidant effects and inhibition of platelet aggregation [23-27], which is consistent with our clinical findings.

There are several possible mechanisms to explain the link between areca nut chewing, CAD and atherosclerosis. Atherosclerosis, especially CAD, was related to chronic inflammation in previous studies [20,28]. Arecoline, the most well-known content of the areca nut, was reported to induce COX-2 up-regulation and higher TIMP expression in *in-vitro *studies [29,30]. Hydroxychavicol, another major phenolic compound in the inflorescence, could induce reactive oxygen species production via redox cycling [31], increase superoxide dismutase activity in mice liver [32] and reduce glutathione in cell line studies [33,34]. One study, done by Lee et al., reported that betel-quid could increase PKC and NF-κB expressions in mice [35]. Subsequently, Ni et al. found that human buccal mucosa cells exposed to areca nut could activate NF-κB expression [36]. The expressions of tumor necrosis factor-alpha, interleukin (IL)-1 beta, IL-6 and IL-8 were also increased in human peripheral blood mononuclear cells after being treated with areca nut extract [37]. The extracts of areca nut, piper inflorescence and betel quid were found to enhance the cytotoxic effects of oxidized low-density lipoprotein (LDL) toward bovine aortic endothelial cells [23]. Because cigarette smoking might enhance the oxidation of LDL cholesterol and lead to atherosclerosis, the enhancement of cytotoxic effects of oxidized LDL by arecoline might explain the additive effect of areca nut chewing and smoking on the risk of CAD.

There were several limitations in the study. The healthy controls were drawn from healthy volunteers attending general health check-ups; thus, this group may not be representative of the general population. The detailed lifestyle information about vegetable consumption and exercise status was not routinely collected in our questionnaire, which might influence the risk of CAD studied here.

## Conclusions

In conclusion, we have shown that long-term areca nut chewing is an independent risk factor of CAD in Taiwanese men, especially among subjects who smoke cigarettes. A dose-response effect of areca nut chewing in relation to the risk of CAD was also found in this study. There is an additive effect of areca nut chewing and cigarette smoking, HTN, and dyslipidemia on the risk of CAD. Further studies are needed to clarify the mechanisms of areca nut chewing leading to CAD.

## Abbreviations

BMI: Body mass index; CAD: Coronary artery disease; CAG: Coronary angiography; CI: Confidence intervals; DM: Diabetes mellitus; HTN: Hypertension; IL: Interleukin; LDL: Low-density lipoprotein; MPI: Myocardial perfusion imaging; OR: Odds ratio; SI: Synergism index; SPECT: Single-photon emission computed tomography; Tl-201: Thallium-201.

## Competing interests

The authors declare that they have no competing interests.

## Authors' contributions

TWC, WMT and LWT designed the study; WGJ, LKT, LYH, YHW, CCS, CYT, LTH, SHM, HPC, CKH, DTH, KYC and SSH conducted the research; LCH, WMT and TWC analyzed data; and TWC and WMT wrote the paper. TWC, WMT and LWT had the primary responsibility for the final content. All authors read and approved the final manuscript.

## Pre-publication history

The pre-publication history for this paper can be accessed here:

http://www.biomedcentral.com/1471-2458/12/162/prepub

## Supplementary Material

Additional file 1 **Table 1**. Odds ratio for coronary artery disease associated with areca nuts chewing in male smoking subjects. Click here for file

Additional file 2 **Table 2**. Odds ratio for obstructive coronary artery disease associated with areca nut use. Click here for file

Additional file 3 **Table 3**. The additive interaction between conventional risk factors and areca nuts use for coronary artery disease. Click here for file
