# Supplementary material for: Chewing areca nut increases the risk of coronary artery disease in taiwanese men: a case-control study
Source: BMC Public Health. 2012 Mar 7;12:162. doi: 10.1186/1471-2458-12-162 (PMC3372426; doi:10.1186/1471-2458-12-162)
Supplement: Additional file 1 — Table 1. Odds ratio for coronary artery disease associated with areca nuts chewing in male smoking subjects. [file 1471-2458-12-162-S1.DOCX]

**Table 1. Odds ratio for coronary artery disease associated with areca nuts chewing in male smoking subjects.**

| **Smoking male** | **Healthy controls**  **(n = 306)** | **Obstructive CAD**  **(n = 217)** | **Crude OR (95% CI)** | | **Adjusted OR (95% CI)*^a^*** | |
| --- | --- | --- | --- | --- | --- | --- |
|  | N (%) | N (%) |  | |  |  |
| Areca nuts |  |  |  | |  |  |
| Never-user | 253 (82.7) | 132 (60.8) | 1.0 |  | 1.0 |  |
| User | 53 (17.3) | 85 (39.2) | 3.2 | (2.1-4.8) | 4.3 | (2.3-7.8) |
| Former user | 32 (10.4) | 67 (30.9) | 1.8 | (0.9-3.4) | 3.7 | (1.4-9.5) |
| Current user | 21 (6.9) | 18 (8.3) | 4.1 | (2.5-6.5) | 4.6 | (2.3-9.0) |
| Daily uses (pieces/day) |  |  |  |  |  |  |
| 1- 20 | 36 (11.8) | 35 (16.1) | 1.9 | (1.1-3.2) | 2.3 | (1.1-4.7) |
| > 20 | 17 (5.5) | 50 (23.1) | 6.1 | (3.3-11.1) | 10.5 | (4.4-25.1) |
| Cumulative uses  (pack-years) |  |  |  |  |  |  |
| 1 – 20 | 33 (10.8) | 30 (13.8) | 1.7 | (1.0-2.9) | 2.3 | (1.1-5.0) |
| > 20 | 20 (6.5) | 55 (25.4) | 5.9 | (3.4-10.5) | 8.0 | (3.6-17.9) |
| Type of uses*^b^* |  |  |  |  |  |  |
| Betel leaf | 34 (11.1) | 36 (16.6) | 2.2 | (1.3-3.7) | 3.4 | (1.5-7.4) |
| Lao-hwa*^,^* | 10 (3.3) | 25 (11.5) | 4.4 | (2.1-9.3) | 6.2 | (2.2-17.2) |
| Both *^c^* | 7 (2.3) | 21 (9.7) | 5.8 | (2.4-14.1) | 3.7 | (1.2-11.5) |
|  |  |  |  |  |  |  |

BMI, body mass index; CAD, coronary artery disease; CI, confidence interval; OR, odds ratio;

*^a^*Adjusting for diabetes, hypertension, dyslipidemia, alcohol drinking, age, educational levels, and BMI. *^b^*Five missing data. *^c^* Lao-hwa and Betel leaf.
