# Supplementary material for: Chewing areca nut increases the risk of coronary artery disease in taiwanese men: a case-control study
Source: BMC Public Health. 2012 Mar 7;12:162. doi: 10.1186/1471-2458-12-162 (PMC3372426; doi:10.1186/1471-2458-12-162)
Supplement: Additional file 2 — Table 2. Odds ratio for obstructive coronary artery disease associated with areca nut use. [file 1471-2458-12-162-S2.DOCX]

**Table 2. Odds ratio for obstructive coronary artery disease associated with areca nut use.**

| **Total** | | **Healthy controls**  **(n = 720)** | | **Obstructive CAD**  **(n = 293)** | **Crude OR (95% CI)** | | **Adjusted OR (95% CI)*^a^*** | |
| --- | --- | --- | --- | --- | --- | --- | --- | --- |
|  | | N (%) | | N (%) |  | |  |  |
| Areca nuts | |  | |  |  |  |  |  |
| Never-user | | 663 (92.1) | | 205 (70.0) | 1.0 |  | 1.0 |  |
| User  Diabetes | | 57 (7.9) | | 88 (30.0) | 5.0 | (3.5-7.2) | 3.5 | (2.1-6.4) |
| No | | 638 (88.6) | | 178 (60.8) | 1.0 |  | 1.0 |  |
| Yes | | 82 (11.4) | | 115 (39.2) | 5.0 | (3.6-7.0) | 2.6 | (1.7-4.2) |
| Hypertension | |  | |  |  |  |  |  |
| No | | 576 (80.0) | | 74 (25.3) | 1.0 |  | 1.0 |  |
| Yes | | 144 (20.0) | | 219 (74.7) | 11.8 | (8.6-16.3) | 10.2 | (6.6-15.8) |
| Dyslippidemia | |  | |  |  |  |  |  |
| No | | 610 (84.7) | | 89 (30.4) | 1.0 |  | 1.0 |  |
| Yes | | 110 (15.3) | | 204 (69.6) | 12.7 | (9.2-17.5) | 13.0 | (8.5-19.9) |
| Alcohol drink (drink-years) | |  | |  |  |  |  |  |
| Never user | | 518 (71.9) | | 191 (65.2) | 1.0 |  | 1.0 |  |
| User 1-20 | | 100 (13.9) | | 46 (15.7) | 1.2 | (0.8-1.8) | 1.0 | (0.5-1.7) |
| > 20 | | 100 (14.2) | | 56 (19.1) | 1.5 | (1.0-2.1) | 0.8 | (0.5-1.5) |
|  | |  | |  |  |  |  |  |
| Cigarette smoking (pack-years) | |  | |  |  |  |  |  |
| Never user | | 414 (57.5) | | 76 (25.9) | 1.0 |  | 1.0 |  |
| User 1-20 | | 133 (18.5) | | 63 (21.5) | 2.6 | (1.8-3.8) | 2.9 | (1.6-5.2) |
| > 20 | | 173 (24.0) | | 154 (52.6) | 4.8 | (3.5-6.7) | 4.8 | (2.9-8.0) |
|  |  | |  | |  | |  | |

BMI, body mass index; CAD, coronary artery disease; CI, confidence interval; OR, odds ratio;

*^a^*Adjusting for age, educational levels, BMI and other covariates in the table.
