# Supplementary material for: Chewing areca nut increases the risk of coronary artery disease in taiwanese men: a case-control study
Source: BMC Public Health. 2012 Mar 7;12:162. doi: 10.1186/1471-2458-12-162 (PMC3372426; doi:10.1186/1471-2458-12-162)
Supplement: Additional file 3 — Table 3. The additive interaction between conventional risk factors and areca nuts use for coronary artery disease. [file 1471-2458-12-162-S3.DOCX]

**Table 3. The additive interaction between conventional risk factors and areca nuts use for coronary artery disease.**

| **Factors/Category** | **Healthy controls (n=720)**  N (%) | | **Obstructive CAD**  **(n=293)**  N (%) | **Crude OR (95% CI)** | | **Adjusted OR (95% CI)** *^a^* | |
| --- | --- | --- | --- | --- | --- | --- | --- |
| Smoke/Areca nuts use |  | |  |  |  |  |  |
| Never/Never | 410 (56.9) | | 73 (24.9) | 1.0 |  | 1.0 |  |
| Ever/Never | 253 (35.1) | | 132 (45.1) | 2.9 | (2.1 – 4.1) | 3.8 | (2.4 – 6.1) |
| Never/Ever | 4 (0.6) | | 3 (1.0) | 4.2 | (0.9 – 19.2) | 1.5 | (0.2 – 13.5) |
| Ever/Ever | 53 (7.4) | | 85 (29.0) | 9.0 | (5.9 – 13.8) | 15.0 | (7.8 – 28.9) |
|  |  | |  |  |  |  |  |
| Diabetes |  | |  |  |  |  |  |
| No | 638 (88.6) | | 178 (60.8) | 1.0 |  | 1.0 |  |
| Yes | 82 (11.4) | | 115 (39.2) | 5.0 | (3.6 – 7.0) | 2.6 | (1.7 – 4.2) |
|  |  | |  |  |  |  |  |
| Hypertension |  | |  |  |  |  |  |
| No | 576 (80.0) | | 74 (25.3) | 1.0 |  | 1.0 |  |
| Yes | 144 (20.0) | | 217 (74.7) | 11.8 | (8.6 – 16.3) | 10.5 | (6.8 – 16.3) |
|  |  | |  |  |  |  |  |
| Dyslipidemia |  | |  |  |  |  |  |
| No | 610(84.7) | | 89(30.4) | 1.0 |  | 1.0 |  |
| Yes | 110(15.3) | | 204(69.6) | 12.7 | (9.2 – 17.5) | 12.7 | (8.3 – 19.5) |
|  |  | |  |  |  |  |  |
| Alcohol drink |  | |  |  |  |  |  |
| Never user | 518 (71.9) | | 191 (65.2) | 1.0 |  | 1.0 |  |
| Ever user | 202(28.1) | | 102 (34.8) | 1.4 | (1.0 – 1.8) | 0.9 | (0.6 – 1.4) |
|  | |  |  |  |  |  |  |

BMI, body mass index; CAD, coronary artery disease; CI, confidence interval; OR, odds ratio;

*^a^*Odds ratios were adjusted for age, educational levels, BMI, and other covariates in the table.
